# Supplementary material for: A New MiRNA MiRm0002 in Eggplant Participates in the Regulation of Defense Responses to Verticillium Wilt
Source: Plants (Basel). 2021 Oct 23;10(11):2274. doi: 10.3390/plants10112274 (PMC8622893; doi:10.3390/plants10112274)
Supplement: Supplementary file 1 [file plants-10-02274-s001.zip › plants-1420630-supplementary.pdf]

Table S1. Primers used in PCR reactions

primer name	primer sequences	function
miRm0002_preF	CATGCCATGGGCCCACAGTACTTGTGAAG	gene clone for miRm0002 precursor
miRm0002_preR	CGGACTAGTTGCCACCTTTTCCTG	
miRm0002_anti-F	CATGCCATGGTGCCACCTTTTCCTG	gene clone for antisense miRm0002 precursor
miRm0002_anti-R	CGGACTAGTGCCCACAGTACTTGTGAAG	
miRm0002_RT	GTCGTATCCAGTGCAGGGTCCGAGGTATTCG CACTGGATACGATGCTAT	Reverse transcription for miRm0002
GFP_R	TTCTGCTTGTCGGCCATGAT	vector-specific reverse primer for molecular identification
miRm0002_F	GCTTGGCTTGTGAAGGTAG	stem-loop qRT-PCR
Uni_R	GTGCAGGGTCCGAGGT	
U6_F	CGGGGACATCCGATAAAA	qRT-PCR as internal reference of *miRm0002*
U6_R	TTGGACCATTTCTCGATTTG	
ARF8_F	TTTCCTCTTTACTGCCACCTT	qRT-PCR
ARF8_R	GGGATACTCATTGCCCACA	
EF-1α_F	ATTCAAGTATGCCTGGGTGCT	qRT-PCR as internal reference of *ARF8*
EF-1α_R	GTGGTGGAGTCAATAATGAGGAC	
ITS_F	CCGCCGGTCCATCAGTCTCTCTGTTTATAC	quantification for DNA level of ITS
ITS_R	CGCCTGCGGGACTCCGATGCGAGCTGTAAC	
α-tublin_F	CCACCATCAAGACTAAGCG	internal standards for ITS DNA level
α-tublin_R	AGAAGACCTCAGCAACACTC	
